# p-Cresyl Sulfate Predicts Ischemic Stroke among Patients on Hemodialysis: A Prospective Cohort Study

**DOI:** 10.1155/2022/1358419

**Published:** 2022-01-31

**Authors:** Xiao Tan, Jianzhou Zou, Fangfang Xiang, Pan Zhang, Bo Shen, Yaqiong Wang, Xiaoqiang Ding, Xuesen Cao

**Affiliations:** ^1^Department of Nephrology, Zhongshan Hospital, Fudan University, No. 180, Fenglin Road, Shanghai, China; ^2^Dialysis Center, Department of Nephrology, Zhongshan Hospital Fudan University, No. 180, Fenglin Road, Shanghai, China; ^3^Shanghai Key Laboratory of Kidney and Blood Purification, No. 180, Fenglin Road, Shanghai, China; ^4^Department of Nephrology, Zhongshan Hospital Fudan University; Shanghai Institute of Kidney and Dialysis, China

## Abstract

**Method:**

Patients on hemodialysis over 6 months were enrolled in this prospective cohort study and were divided into 2 groups based on plasma p-cresyl sulfate level. The primary end point was the first episode of ischemic stroke during follow-up. The association between p-cresyl sulfate and ischemic stroke incidence was analyzed by Kaplan-Meier method and Cox proportional hazard model.

**Results:**

220 patients were enrolled in this study. 44 patients experienced episodes of first ischemic stroke during follow-up for 87.8 (47.6-119.5) months. Kaplan-Meier analysis demonstrated that the incidence of ischemic stroke in the high p-cresyl sulfate group was significantly higher than that in the low p-cresyl sulfate group (Log-Rank *P* = 0.007). Cox regression analysis as well proved that p-cresyl sulfate level was significantly associated with the first incidence of ischemic stroke (HR (hazard ratio) 2.332, 95% CI (95% confidence interval) 1.236-4.399, *P* = 0.009). After being adjusted for other confounding risk factors, the results persisted significant (model 11: HR 2.061, 95% CI 1.030-4.125, *P* = 0.041).

**Conclusion:**

Plasma p-cresyl sulfate predicts the first incidence of ischemic stroke in hemodialysis patients.

## 1. Introduction

The relationship between kidney diseases and cerebrovascular diseases has become increasingly recognized in recent years. The incidence of cerebrovascular disease is higher among chronic kidney disease (CKD) patients compared to that in the healthy population, and the prevalence of cerebrovascular disease is higher in more advanced stages of CKD [1]. CKD patients, especially end-stage renal disease (ESRD) patients, are with increased hospitalization rates [2, 3] and mortality [4] associated with ischemic stroke.

During CKD progression, uremic toxins accumulate in the circulation since kidney function declines. Among the uremic toxins, protein-bound uremic toxins have recently been noted as a potential link in cardiorenal syndrome [5], and removal of protein-bound uremic toxins by dialysis is extremely difficult due to their high protein-binding affinity [6]. This has been well demonstrated with two of the most typical protein-bound uremic toxins: *p*-cresyl sulfate (PCS) [7–10] and indoxyl sulfate (IS) [11–13]. Our past research demonstrated that plasma indoxyl sulfate was associated with the first heart failure event in patients on hemodialysis [14]. In this study, we focused on the other protein-bound uremic toxin, PCS.

PCS, with a molecular weight of 188.2 g/mol, originates from sulfation (para-) of the intestinally generated *p*-cresol, and it is bound to about 95% to the protein albumin in the circulation [15]. In normal condition, the clearance value of indoxyl sulfate is 1055 ± 148 mL/min/1.73 m^2^, which is 8 ± 1 times of creatinine [16], but it increases significantly in uremic patients (uremic patients 20.9 ± 12.2 *μ*g/mL vs. normal 1.9 ± 1.3 *μ*g/mL) due to renal dysfunction [17]. And plasma levels of PCS were increased with even moderate impairment of renal function [18]. In the past decade, a growing number of publications documented the impact of PCS on CKD progression, cardiovascular diseases, and mortality [7–10, 19]. An existing study also suggests that p-cresyl sulfate is a significant independent predictor of carotid plaque burden [20]. However, the clinical association of PCS and stroke is uncertain. We, therefore, conducted the current prospective study to investigate the relationship between PCS and ischemic stroke in hemodialysis patients.

## 2. Materials and Methods

### 2.1. Study Population and Endpoint Evaluation

The study population consisted of 220 patients ≥ 18 years old, who underwent regular hemodialysis therapy over 6 months in the Blood Purification Center, Zhongshan Hospital, Fudan University. The enrollment was completed within 6 months from July to December 2009. The patients who had heart failure and acute myocardial infarction within 3 months before the study, as well as who had stroke ever, were excluded from our study. Patients were treated thrice weekly (4 hours per session). The study was performed according to the Declaration of Helsinki and approved by the Ethical Committee, Zhongshan Hospital, Fudan University. All participants provided written informed consent.

Ischemic stroke was defined according to ICD-9 diagnosis codes by 2 physicians according to brain imaging (computed tomography and/or magnetic resonance imaging). The primary endpoint was the first incidence of ischemic stroke. The secondary endpoint was death, kidney transplantation, and transfer to other dialysis centers.

### 2.2. Anthropometric Measurements, Biochemical Measurements, and Clinical Data Collection

Demographic and clinical data includes age, sex, dialysis duration, smoking history, history of medicine application, underlying kidney disease, and comorbidities. Height and weight were measured while patients were without shoes and with light clothes. Body mass index (BMI) was calculated according to the following formula: weight (in kg)/height^2^ (in m^2^). Blood pressure was defined as the average of all predialysis blood pressure during 4 weeks (12 times in total) before this study. Blood sampling was achieved during a midweek nondialysis day 8-10 am. Serum blood urea nitrogen (BUN), serum creatinine (SCr), hemoglobin, albumin, pre-albumin, calcium (Ca), phosphorus (P), lipids, uric acid (UA), total homocysteine (tHcy), iron, transferrin, and ferritin were measured via standard methods by the clinical laboratory. The concentrations of high-sensitivity C-reactive protein (hsCRP) and *β*_2_-microglobulin (*β*_2_M) were measured by immunoturbidimetry assay, and the concentration of iPTH (intact parathyroid hormone) was measured by electrochemiluminescence immunoassay.

### 2.3. PCS Measurement

Standard of PCS (99.8%) was kindly provided by Professor Raymond Vanholder (Ghent University Hospital). Internal standard of warfarin (99.5%) was kindly provided by Shanghai Institute for Drug Control. High-performance liquid chromatography tandem mass spectrometry (HPLC-MS/MS) method was used to detect PCS concentration in plasma. Briefly, 100 *μ*L plasma was pipetted to a 1.5 mL polypropylene tube. Then, 500 *μ*L of internal standard/protein precipitation solution (50 ng/mL warfarin in methanol) was added to precipitate the proteins. The contents were vortex mixed for 1 min. After centrifugation at 12 000 × g for 10 min, a 100 *μ*L aliquot of clear supernatant was mixed with 100 *μ*L of water in a polypropylene tube and transferred to an autosampler. A volume of 5 *μ*L was injected into LC-MS/MS. The chromatographic separation was achieved on a Venusil XBP Phenyl column (100 mm × 2.1 mm, 5 *μ*m; Bonna-Agela Technologies Inc, Wilmington, DE, USA). Mobile phase A was 2 mmol/L ammonium acetate in 0.1% formic acid (*v*/*v*). Mobile B was methanol. The mobile phase (A : B = 30 : 70) was delivered at a flow rate of 0.35 mL/min. The temperature of the column and autosampler was maintained at 40°C and 4°C, respectively. Mass spectrometric detection was performed on an API 3000 triple quadrupole instrument (Applied Biosystems, Toronto, ON, Canada) in multiple reaction monitoring (MRM) mode. A TurboIonSpray ionization (ESI) interface in negative ionization mode was used. Turbo spray voltage was set at -4200 V. Source temperature was maintained at 500°C. The compound parameters, collision energy (CE), declustering potential (DP), entrance potential (EP), and collision exit potential (CXP) were -27 V, -30 V, -10 V, and -15 V for PCS and -20 V, -46 V, -10 V, and -15 V for warfarin. Quadrupole 1 and quadrupole 3 were maintained at unit resolution. Dwelling time set was 200 ms for all the analytes. Mass transitions *m*/*z*187.1⟶107.1 for PCS and *m*/*z*307.0⟶249.7 for warfarin were used. Data processing was performed with Analyst 1.4.1 software package (Applied Biosystems, Toronto, ON, Canada). Standard curve for IS was set at 0.025, 0.05, 0.1, 0.5, 1, 5, 10, and 40 *μ*g/mL, with an average r value of 0.999 (*n* = 8). The lower limit of quantitation was 0.025 *μ*g/mL. Data analysis was performed with Analyst 1.4.1 software package (Applied Biosystems, Toronto, ON, Canada).

### 2.4. Statistical Analyses

For the primary endpoint, the Kaplan-Meier method and Cox proportional hazard model were used to evaluate the association between PCS and the first incidence of ischemic stroke. To adjust confounding risk factors, we constructed Model 1 (age, sex, and BMI), Model 2 (hemoglobin, iron, transferrin, and ferrintin), Model 3 (history of smoking, primary hypertension, coronary heart disease, diabetes, and uarthritis), Model 4 (systolic blood pressure (SBP), diastolic blood pressure (DBP), urinary volume, and single-pool Kt/V (spKt/V)), Model 5 (albumin, prealbumin, BUN, SCr, UA, and glucose), Model 6 (triglyceride (TG), total cholesterol (TC), low-density lipoprotein cholesterol (LDL-C), high-density lipoprotein cholesterol (HDL-C), apolipoprotein A (Apo-A), apolipoprotein B (Apo-B), and tHcy), Model 7 (Ca, P, iPTH, and 25 hydroxyl vitamin D (25OHvitD)), Model 8 (hsCRP and *β*_2_M), Model 9 (history of taking calcium channel entry blockers (CCB), angiotensin-converting enzyme inhibitors (ACEI), angiotensin receptor blockers (ARB), *β*-blocker, *α*-blocker, aspirin, statin, calcium-based phosphate binders, and 1,25(OH)2vitD3), Model 10 (N-terminal probrain natriuretic peptide (NT-proBNP), Left Ventricular Mass Index (LVMI), and left ventricular ejection fraction (LVEF)), and Model 11. The criterion for Model 11 selection was determined as *P* < 0.05 in the univariate Cox proportional hazard model. PCS was entered as a dichotomous variable.

All data were expressed as mean ± SD, median (or interquartile range), or frequency, as appropriate. To compare the two groups of normal data, an independent samples *t*-test was conducted. A two-tailed *P* < 0.05 was considered statistically significant. All data analyses were performed via SPSS 22.0 (SPSS Inc., Chicago, IL, USA).

## 3. Results

### 3.1. Baseline Characteristics of the Study Population

The baseline characteristics of the patients are listed in Table 1. The cohort consisted of 220 hemodialysis patients (125 males), with an age of 56 ± 14 years. Glomerular disease was the leading cause of end-stage renal disease, accounting for 44.1%. The prevalence of primary hypertension, CHD (coronary heart disease), diabetes, and uarthritis was 27.7%, 5.9%, 11.4%, and 22.7%, respectively. According to the plasma PCS concentration, patients were categorized into two groups: low-PCS group (PCS ≤ 20.10 *μ*g/mL) and high-PCS group (PCS > 20.10 *μ*g/mL). Compared with the low-PCS group, patients in the high-PCS group had lower ARB and 1,25(OH)2vitD3 medication rate and lower serum hsCRP (high-sensitivity C-reactive protein), as well as higher serum albumin and creatine. There were no significant differences in other characteristics (Table 1).

Median follow-up time was 87.8 (47.6-119.5) months. During follow-up, 44 patients experienced episodes of ischemic stroke, and 5 of which were followed by cerebral hemorrhage. 10 patients had acute myocardial infarction. 16 patients were lost to follow-up because of transference to a different center. 15 patients received kidney transplantation. 101 patients died, of which 9 were classified as death caused by ischemic stroke, 6 as death caused by cerebral hemorrhage, and 25 as cardiac death.

### 3.2. Association between Serum p-Cresyl Sulfate Level and Ischemic Stroke

**Figure 1:**
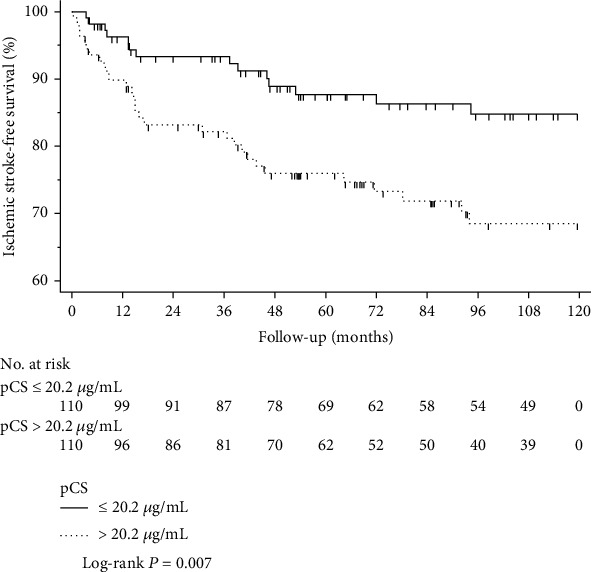
Kaplan-Meier curves of first incidence of ischemic stroke during follow-up in hemodialysis patients stratified by the low- and high-PCS group.

In this study, 44 patients experienced the first incidence of ischemic stroke. In the crude analysis by the Kaplan-Meier method, we found that the incidence of ischemic stroke in the high-PCS group was significantly higher than that in the low-PCS group (Log-Rank *P* = 0.007) (Figure 1).

**Figure 2:**
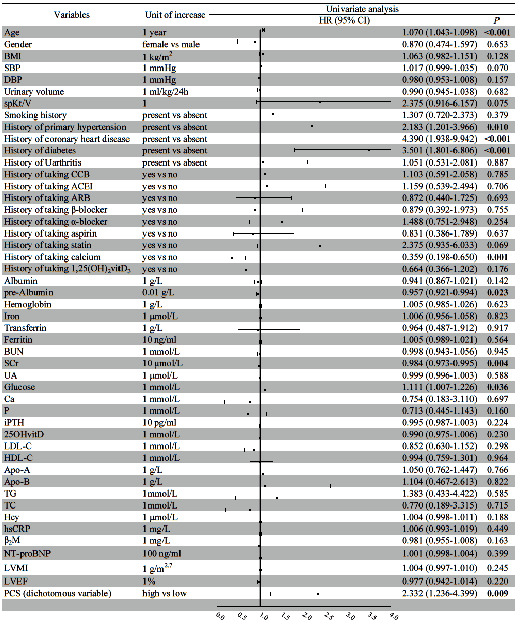
Univariate Cox hazard ratios for the incidence of first ischemic stroke.

In the univariate Cox proportional hazard model, PCS was entered only as a dichotomous variable. Results showed that PCS was significantly associated with first cerebral infarction (HR 2.332, 95% CI 1.236-4.399, *P* = 0.009) (Figure 2). A series of models were constructed to adjust confounding risk factors, including Models 1-11. PCS was still significant in Models 1-10(Table 2). In Model 11 (hierarchically selected covariates of age, serum prealbumin, SCr, serum glucose, history of primary hypertension, history of coronary heart disease, history of diabetes, and history of taking calcium-based phosphate binders), result still remained significant after adjustment for confounding risk factors listed above (HR 2.061, 95% CI 1.030-4.125, *P* = 0.041) (Table 3). In Model 11, age, history of diabetes, and history of taking calcium-based phosphate binders as well are associated with ischemic stroke after adjustment of other confounding risk factors (Table 3).

## 4. Discussion

Hemodialysis patients face a higher risk and poorer outcomes of ischemic stroke, due to special risk factors in this particular population. However, stroke prevention measures in patients on dialysis remain similar to those in general population, and treatment options for reducing ischemic stroke in hemodialysis patients remain limited. It is critical to identify particular risk factors for stroke in ESRD, to develop novel prevention measures and treatment strategies.

In this prospective cohort study, we found that a protein-bound uremic toxin, p-cresyl sulfate, predicts the incidence of newly developed ischemic stroke in hemodialysis patients. PCS is a kind of protein-bound uremic toxin originating from intestinally generated *p*-cresol. Existing studies focused on the relationship between serum PCS level and mortality, especially cardiovascular mortality in the hemodialysis [7, 21] and CKD patients [10, 22].

Our study innovatively provided evidence for an association between higher serum PCS level and an increased risk of ischemic stroke in hemodialysis patients. Result still remained significant after adjustment for other risk factors, suggesting that PCS is independently associated with the first incidence of ischemic stroke in hemodialysis patients.

Endothelial dysfunction is one possible explanation for the association between high serum PCS and ischemic stroke. Meijers et al. [23] found that serum *p*-cresol concentration is independently associated with the number of circulating EMPs (endothelial microparticles, surrogate biomarkers for endothelial dysfunction, and also could be biomarkers of ischemic [24] and hemorrhagic [25] stroke) in hemodialysis patients, and PCS induces EMP shedding in vitro. Cell experiments demonstrated that PCS activates leucocyte [26], human vascular smooth muscle cells, and human umbilical vein endothelial cell [27] free radical production, promoting both vascular dysfunction and vascular remodeling. PCS also exerts proinflammatory effects that contribute to vascular damage by motivating the crosstalk between leukocytes and vessels [28]. Endothelial damage is an essential cause of ischemic stroke. Once the endothelium is impaired, arterial smooth muscle cells proliferate and lead to further contraction of the vessel lumen. Mast cells release elastase and metalloproteinases, contributing to eventual plaque rupture and stroke [29]. Endothelium injury plays important roles in the development of cerebral hemorrhage. Brain endothelial cells function in the maintenance of the blood–brain barrier [30], of which integrity disrupts during and after hemorrhage. Endothelial cells also participate in the delayed phase of hemorrhage, including cerebral vasospasm, microthrombosis, and inflammation, affecting its prognosis [31].

We also identified other risk factors for stroke besides serum PCS level, including age, history of diabetes, and history of taking calcium-based phosphate binders. Diabetes mellitus is a risk factor for stroke in the general population [32]; our result suggests that diabetes may also be a risk factor of ischemic stroke in hemodialysis patients. Calcium-based phosphate binders are widely used in hemodialysis patients with hyperphosphatemia [33]. Although there are few clinical evidences indicating that calcium load directly leads to vascular calcification, a clinical trial suggests that non-calcium-containing phosphate binder such as sevelamer may contribute to lower vascular calcification compared with calcium-containing binders [34]. However, in the year of 2009, non-calcium-containing phosphate binders were not used in our center.

Our study has several strengths, including long follow-up time, prospectively collected data, and that a series of possible confounders were adjusted for. Many studies suggest that PCS contributes to endothelial damage and vascular remodeling, while no clinical evidence ever demonstrated the association between PCS and stroke. Our study first demonstrated that PCS is associated with the first incidence of ischemic stroke. The limitation of our study is that a single time point of serum PCS measurement may not appropriately describe intraindividual variability in levels over time and thus may lead to misclassification of patients into appropriate categories.

## 5. Conclusions

In summary, we demonstrated that high plasma PCS level was associated with higher risk of first incidence of ischemic stroke in hemodialysis patients. Our finding was independent of a series of conventional and unconventional risk factors. Our results suggest that PCS may be an important biomarker to predict ischemic stroke in a hemodialysis population.

## Figures and Tables

**Table 1:** Baseline demographic, clinical, and biochemical characteristics.

	All patients (*n* = 220)	Low-PCS group (PCS ≤ 20.10 *μ*g/mL) (*n* = 110)	High-PCS group (PCS > 20.10 *μ*g/mL) (*n* = 110)	*P*
Age (year)	56 ± 14	55 ± 13	57 ± 16	0.093
Sex (M/F)	125/95	59/51	66/44	0.207
Height (m)	1.56 ± 0.09	1.64 ± 0.09	1.65 ± 0.09	0.973
Weight (kg)	58.7 (52.0, 66.2)	57.6 (52.3, 65.6)	60.3 (48.8, 69.3)	0.799
BMI (kg/m^2^)	21.8 (19.9, 24.0)	21.8 (20.0, 23.7)	21.7 (19.2, 24.0)	0.595
SBP (mmHg)	136 ± 17	136 ± 17	137 ± 17	0.656
DBP (mmHg)	82 ± 10	83 ± 10	82 ± 10	0.295
spKt/V	1.34 (1.17, 1.59)	1.33 (1.10, 1.53)	1.36 (1.20, 1.66)	0.011
Urinary volume (mL/kg/24 h)	0 (0, 5.80)	0 (0, 5.60)	1.09 (0, 5.93)	0.596
Smoking history (%)	36.4	28.2	44.5	0.017
Underlying kidney disease				0.359
Glomerular disease (%)	44.1	40.9	47.3	
Diabetic nephropathy (%)	7.3	7.3	7.3	
Hypertensive nephropathy (%)	7.3	8.2	6.4	
Polycystic kidney disease (%)	6.8	10	3.6	
Medicinal nephropathy (%)	4.5	6.4	2.7	
Others (%)	12.3	10.9	13.6	
Unknown (%)	17.7	16.4	19.1	
Comorbidity				
Primary hypertension (%)	27.7	26.4	29.1	0.382
CHD (%)	5.9	3.6	8.2	0.126
Diabetes (%)	11.4	8.2	14.5	0.101
Uarthritis (%)	22.7	24.5	20.9	0.315
Medications				
CCB (%)	62.3	64.5	60	0.289
ACEI (%)	15.9	12.7	19.1	0.134
ARB (%)	26.8	33.6	20.0	0.033
*β*-Blocker (%)	17.3	20	14.5	0.186
*α*-Blocker (%)	20.0	21.8	18.2	0.307
Aspirin (%)	20.5	20	21.0	0.514
Statin (%)	5.9	5.5	6.4	0.500
Calcium (%)	67.3	70.0	64.5	0.236
1,25(OH)_2_vitD_3_ (%)	54.5	62.7	46.4	0.011
Albumin (g/L)	40 (37, 42)	39 (36, 41)	40 (38, 42)	0.010
Prealbumin (g/L)	0.34 ± 0.08	0.33 ± 0.08	0.35 ± 0.08	0.736
Hemoglobin (g/L)	104 (96, 113)	104 (94, 112)	106 (97, 114)	0.258
Iron (*μ*mol/L)	10.8 (7.8, 15.0)	10.6 (6.8, 14.7)	11.3 (8.5, 15.5)	0.070
Transferrin (g/L)	1.90 (1.65, 2.15)	1.89 (1.66, 2.19)	1.93 (1.64, 2.12)	0.859
Ferritin (ng/mL)	121 (68.8, 260.7)	113.0 (61.3, 263.6)	126.2 (68.9, 258.1)	0.724
BUN (mmol/L)	23.9 ± 5.3	23.2 ± 5.0	24.5 ± 5.6	0.275
SCr (*μ*mol/L)	1004 (863, 1206)	980 (855, 1113)	1030 (887, 1273)	0.042
UA (*μ*mol/L)	433 (382, 494)	428 (382, 483)	439 (377, 500)	0.530
Glucose (mmol/L)	5.4 (4.4, 6.7)	5.5 (4.4, 6.7)	5.3 (4.4, 6.8)	0.841
25OHvitD (nmol/L)	57.3 ± 18.9	56.1 ± 19.3	58.4 ± 18.5	0.578
Ca (mmol/L)	2.20 ± 0.21	2.20 ± 0.21	2.22 ± 0.21	0.866
P (mmol/L)	2.17 ± 0.63	2.27 ± 0.62	2.06 ± 0.63	0.689
iPTH (pg/mL)	276.9 (136.9, 559.4)	289.3 (144.3, 587.5)	270.1 (136.7, 518.8)	0.274
hsCRP (mg/L)	2.0 (0.7, 6.1)	2.8 (0.7, 9.0)	1.4 (0.7, 4.3)	0.038
TG (mmol/L)	1.44 (1.08, 1.98)	1.36 (1.07, 1.90)	1.46 (1.08, 2.01)	0.510
TC (mmol/L)	4.25 (3.72, 5.00)	4.16 (3.70, 4.86)	4.33 (3.72, 5.26)	0.420
HDL-C (mmol/L)	1.10 (0.89, 1.37)	1.11 (0.92, 1.39)	1.06 (0.86, 1.35)	0.216
LDL-C (mmol/L)	2.42 (1.87, 2.95)	2.40 (1.89, 2.90)	2.45 (1.86, 3.09)	0.594
Apo-A (g/L)	1.18 (1.02, 1.40)	1.22 (1.03, 1.44)	1.15 (1.01, 1.35)	0.172
Apo-B (g/L)	0.81 (0.69, 0.98)	0.80 (0.69, 0.98)	0.83 (0.69, 0.99)	0.578
Lp(a) (mg/L)	175.5 (114.0, 280.8)	175.0 (121.0, 302.5)	175.5 (109.8, 273.3)	0.716
tHcy (*μ*mol/L)	35.4 (28.0, 45.7)	33.3 (27.2, 44.8)	36.8 (30.3, 45.9)	0.074
*β*_2_M (mg/L)	36.1 (30.3, 42.7)	36.4 (30.2, 43.1)	35.6 (30.6, 41.6)	0.593
NT-proBNP (ng/mL)	3807 (1747, 8816)	3696 (1379, 10352)	4097 (1991, 7917)	0.601
LVMI (g/m^2.7^)	108.2 (90.6, 137.8)	110.1 (90.4, 141.7)	106.8 (90.8, 129.1)	0.529
LVEF (%)	67 (62, 72)	68 (63, 73)	66 (62, 70)	0.158
PCS (*μ*g/mL)	22.52 ± 16.22	9.53 ± 5.38	35.53 ± 12.60	<0.001

Abbreviations: BMI: body mass index; SBP: systolic blood pressure; DBP: diastolic blood pressure; spKt/V: single-pool Kt/V; CHD: coronary heart disease; CCB: calcium channel blocker; ACEI: angiotensin conversion enzyme inhibitor; ARB: angiotensin receptor blocker; BUN:, blood urea nitrogen; SCr: serum creatinine; UA: uric acid; ALP: alkaline phosphatase; Ca: calcium; P: phosphorus; Ca∗P: calcium phosphorus product; iPTH: intact parathyroid hormone; hsCRP: high-sensitivity C-reactive protein; TG: triglyceride; TC: total cholesterol; HDL-C:, high-density lipoprotein cholesterol; LDL-C: low-density lipoprotein cholesterol; Apo-A: apolipoprotein A; Apo-B: apolipoprotein B; Lp(a): lipoprotein (a); tHcy: total homocysteine; *β*_2_M: *β*2-microglobulin; PCS: p-cresyl sulfate.

**Table 2:** Multivariate Cox for the incidence of first ischemic stroke.

	HR	95% CI	*P*
p-Cresyl sulfate (dichotomous variable)			
Unadjusted	2.332	1.236-4.399	0.009
Model 1	1.998	1.041-3.834	0.037
Model 2	2.368	1.246-4.501	0.009
Model 3	1.956	1.008-3.796	0.047
Model 4	1.994	1.034-3.847	0.039
Model 5	3.291	1.707-6.344	<0.001
Model 6	2.504	1.313-4.778	0.005
Model 7	2.319	1.209-4.447	0.011
Model 8	2.313	1.226-4.365	0.010
Model 9	2.155	1.121-4.143	0.021
Model 10	2.343	1.236-2.439	0.009
Model 11	2.061	1.030-4.125	0.041

HR: hazard ratio; 95% CI: 95% confidence interval; Model 1: adjusted for age, sex, and BMI; Model 2: adjusted for hemoglobin, iron, transferrin, and ferrintin; Model 3: adjusted for history of smoking, primary hypertension, coronary heart disease, diabetes, and uarthritis; Model 4: adjusted for SBP, DBP, urinary volume, and spKt/V; Model 5: adjusted for albumin, prealbumin, BUN, SCr, UA, and glucose; Model 6: adjusted for TG, TC, LDL-C, HDL-C, Apo-A, Apo-B, and tHcy; Model 7: adjusted for Ca, P, iPTH, and 25OHvitD; Model 8: adjusted for hsCRP and *β*_2_M; Model 9: adjusted for history of taking CCB, ACEI, ARB, *β*-blocker, *α*-blocker, aspirin, statin, calcium, and 1,25(OH)2vitD3; Model 10: adjusted for NT-proBNP, LVMI, and LVEF; Model 11: hierarchically selected covariates of age, serum prealbumin, SCr, serum glucose, history of primary hypertension, history of coronary heart disease, history of diabetes, and history of taking calcium-based phosphate binders.

**Table 3:** Multivariate Cox for the incidence of first ischemic stroke (Model 11).

Variables	Univariate *P*	Multivariate analysis		
		HR	95% CI	*P*
Age	<0.001	1.051	1.019-1.084	0.002
SCr	0.004	1.000	0.998-1.001	0.829
Glucose	0.036	1.006	0.998-1.001	0.976
Prealbumin	0.023	0.976	0.937-1.017	0.255
History of primary hypertension	0.010	0.990	0.479-2.045	0.978
History of coronary heart disease	<0.001	1.872	0.721-4.861	0.198
History of diabetes	<0.001	2.733	1.221-6.120	0.015
History of taking calcium	0.001	0.440	0.237-0.818	0.010
PCS	0.009	2.061	1.030-4.125	0.041

Abbreviations: SCr: serum creatinine; PCS: p-cresyl sulfate.

## Data Availability

Data used during the study are available from the corresponding author by request.
